# Clinical characteristics associated with peripartum maternal bloodstream infection

**DOI:** 10.3389/fmicb.2024.1454907

**Published:** 2024-11-13

**Authors:** Xiao-Li Gao, Yang Li, Su-Juan Hou, Wen-Jun Fan, Ling-Yi Fang, Shi-Jun Ni, Ye Yan, Jie Li, Cha Han

**Affiliations:** ^1^Tianjin Key Laboratory of Female Reproductive Health and Eugenics, Tianjin Medical University General Hospital, Tianjin, China; ^2^Department of Obstetrics and Gynecology, Tianjin Medical University General Hospital, Tianjin, China

**Keywords:** delivery, peripartum, bloodstream infection, sepsis, risk factors

## Abstract

**Objective:**

Bloodstream infection (BSI) during the peripartum period is a major cause of maternal morbidity and mortality. However, data on maternal BSI during hospitalization for delivery are limited. This study aimed to investigate the incidence, clinical characteristics, risk factors, microbiological features, and antibiotic resistance patterns of maternal peripartum BSI, with a focus on understanding the role of premature rupture of membranes (PROM), fever, and other risk factors in its development.

**Methods:**

We investigated the clinical characteristics associated with maternal BSI during the peripartum period. This study included febrile women with blood cultures obtained during hospitalization for delivery. We analyzed the clinical characteristics, pathogenic microorganisms, antibiotic resistance, and maternal and neonatal outcomes of these patients. Participants were divided into BSI (*n* = 85) and non-BSI (*n* = 361) groups.

**Results:**

Spontaneous rupture of membranes, PROM, PROM >24 h before labor, vaginal examinations >5 times, and cesarean sections during labor were more common in the BSI group. *Escherichia coli* (51.8%; 44/85) was the predominant causative pathogen, followed by *Enterococcus faecalis* (7.1%, 6/85). Approximately 31.2% of *E. coli* were resistant to levofloxacin, and 38.6% were extended-spectrum β-lactamase-producing bacteria. The BSI group had higher rates of maternal sepsis and Apgar scores ≤ 7 at 1 min than the non-BSI group. Furthermore, PROM, fever ≥38.9°C (102°F), and fever within 24 h after delivery were risk factors for postpartum BSI in the adjusted analysis.

**Conclusion:**

Maternal BSI is a potentially life-threatening disease associated with PROM and the timing and severity of fever. Early identification and surveillance of pathogen composition and antimicrobial resistance can help prevent adverse outcomes.

## Introduction

Despite global efforts to improve maternal outcomes, pregnancy-related infection remains a major clinical issue with high morbidity and mortality. The Global Maternal Sepsis Study group reported that infection-related maternal deaths accounted for over half of intrahospital deaths (Aman et al., 2020). Bloodstream infection (BSI) is a common and serious cause of maternal mortality, ranking among the top seven in Europe (Van Dillen et al., 2010) and 11th in the United States (Lapinsky, 2013). Notably, maternal BSI during the peripartum period remains a substantial clinical problem with high morbidity and mortality rates in healthcare (Surgers et al., 2013; Zou et al., 2021). However, data on microbiological characteristics, clinical features, and maternal and fetal prognoses are limited owing to scarce research on maternal BSI.

BSI is a type of sepsis-related infection (Guo et al., 2023) with varied, non-specific symptoms such as fever and chills. However, organ dysfunction occurs once the infection progresses to sepsis (Huerta and Rice, 2019). Furthermore, BSI can be definitively diagnosed using blood culture, and identifying its risk factors is crucial for assessing the risk of adverse outcomes in peripartum women with suspected infections (Easter et al., 2017). Therefore, this retrospective study aimed to investigate the clinical characteristics of peripartum women using blood cultures obtained during hospitalization for delivery. This investigation was conducted with the goal of preventing or reducing the occurrence of BSI and improving maternal and neonatal outcomes from fever onset or earlier. Furthermore, we analyzed microbiological characteristics, antibiotic susceptibility, and risk factors in BSI cases, using maternal BSI as definitive evidence of significant infection, to provide a theoretical basis for preventing peripartum infections.

## Materials and methods

### Study design

This retrospective cohort study of obstetric patients was conducted using blood cultures at Tianjin Medical University General Hospital between January 2018 and August 2023. This study was approved by the Ethical Review Board of our hospital (Approval number: IRB2024-YX-101-01). Overall, 19,157 obstetric patients were screened and enrolled in the study. The criteria for selecting the participants include: (1) patients hospitalized for delivery, (2) having a blood culture (our institution collected blood samples when patients had peripartum fever >38°C [100.4°F] and met one of the following conditions: maternal heart rate exceeding 100 bpm, fetal heart rate exceeding 160 bpm, uterine tenderness, foul-smelling amniotic fluid, or maternal white blood cell count over 15 × 10^9^/L (Mohn et al., 2024), and (3) at least 28 weeks of gestational age up to 7 days after birth. We excluded women with contaminated blood cultures, including coagulase-negative *Staphylococcus* species, *Cutibacterium acnes, Micrococcus, Streptococcus viridans, Corynebacterium, Aerococcus*, or *Bacillus* (Clinical and Laboratory Standards Institute, 2022), and fetal anomalies.

### Data collection

We extracted demographics, clinical characteristics, infection features, laboratory test results, and outcomes from patients' medical records. Demographic and clinical characteristics included age, gravidity, parity, pre-gestational body mass index (BMI), maternal comorbidities, and other obstetric variables. Additionally, the clinical features of being infected included temperature, time of fever, duration of fever, and infection sources. Furthermore, laboratory test results included blood cultures, antibiotic resistance patterns, and microbial testing of other samples. Lastly, maternal and neonatal outcomes included neonatal and maternal and intensive care unit (ICU) admission, maternal sepsis, Apgar scores at 1 and 5 min, neonatal bacteremia or sepsis.

### Blood collection, processing, and antimicrobial susceptibility testing

We aseptically collected two aerobic and anaerobic blood samples (8–10 mL each) from two peripheral veins and incubated them in the Bactec^TM^FX (BD Company, USA) blood culture system. If blood cultures were positive, subcultures were prepared on blood agar plates (Antubio, Zhengzhou, China) in a biosafety cabinet. Subsequently, gram staining was performed, and critical values were reported. The plates were incubated overnight in a Thermo 371 microbiological incubator (Thermo Fisher Scientific, USA). We used the VITEK MS microbial mass spectrometry identification system (bioMérieux, France) to rapidly identify pure colonies and performed susceptibility testing using either the VITEK-2 compact system (bioMérieux, Marcy-l'Étoile, France) or the Phoenix automated microbiological identification system (BD Company, USA).

### Intrauterine collection and identification of microorganisms

Microbial samples were collected using swabs via swirling on the amniotic surface adjacent to the fetal side after ensuring the maternal blood was cleared and carefully avoiding contamination from the maternal side. The samples were cultured on blood and chocolate agar plates. Subsequently, we isolated single suspected colonies from the mixed cultures on the media surface, re-streaked on blood and chocolate agar plates, and incubated at 35°C for 24–48 h in a carbon dioxide incubator. Furthermore, individual colonies were identified after purification and subjected to antimicrobial susceptibility testing using the same methods used for blood samples.

### Definitions

BSI was defined as positive blood cultures in patients showing systemic signs of infection, including primary (without an identified origin) and secondary BSI (Centers for Disease Control and Prevention, 2023). Fever during pregnancy and peripartum was defined as temperature ≥38°C (100.4°F). Furthermore, polymicrobial infections were defined as the isolation of two or more strains from maternal blood cultures. Maternal sepsis is a life-threatening condition defined as organ dysfunction resulting from infections during pregnancy, childbirth, post-abortion, or the postpartum period (World Health Organization, 2017). Organ dysfunction was assessed using the quick Sequential Organ Failure Assessment criteria as follows: systolic blood pressure ≤ 100 mmHg, respiratory rate ≥22 per min, and altered mental status. A score of ≥2 indicated a poor prognosis (Shields et al., 2023). Antibiotics were defined as J01 in the World Health Organization (WHO) anatomical therapeutic chemical/defined daily dose (ATC/DDD) classification (WHO Collaborating Centres, 2024). Prophylactic antibiotic use differs from treatment with antibiotics in that the former is intended to prevent infection, whereas the latter is intended to resolve an established infection, typically requiring a longer course of therapy.

### Statistical analysis

Statistical analyses were performed using the statistical package for social sciences (SPSS) 27.0 (IBM Corp., Armonk, NY, USA). The Chi-square or Fisher's exact test was used for categorical variables, and the Student's *t*-test or Wilcoxon Rank-sum test was used for continuous variables. Statistical significance was set at *p* < 0.05. Logistic regression was used to examine the associations between various exposures and postpartum BSI while controlling for potential confounders. We selected confounders based on their association with the outcomes of interest or a change in the effect estimate >10%. Stepwise backward elimination was performed to create the final model. All variables with *p* < 0.05 in the final model were considered significant independent risk factors for postpartum BSI.

## Results

**Figure 1:**
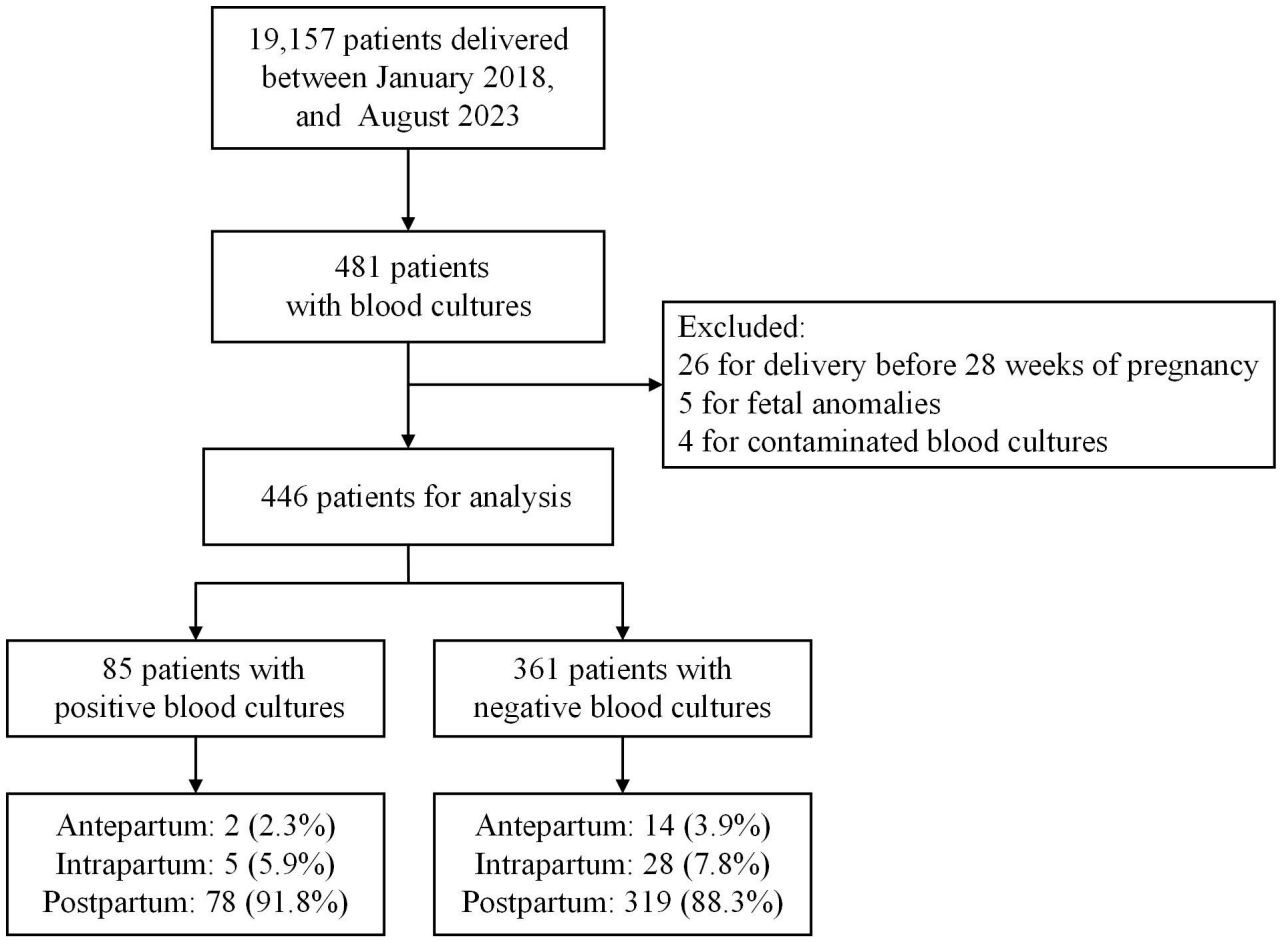
Flow diagram of inclusion criteria, time of blood culture sampling of peripartum women.

Of the 19,157 pregnant women who delivered during hospitalization between January 2018 and August 2023, 35 were excluded: 26 for delivery before 28 weeks of pregnancy, five for fetal anomalies, and four for contaminated blood cultures. In total, 85 and 361 eligible women with positive and negative blood cultures, respectively, were enrolled in the study. Figure 1 presents a flowchart of the study.

### Demographic and clinical characteristics

The demographic and clinical characteristics of eligible patients are presented in Table 1. Age, pre-pregnancy BMI, and the percentage of comorbidities and preterm birth did not significantly differ between the groups (*p* > 0.05). Patients in the BSI group had a higher incidence of spontaneous rupture of membranes (47.1% vs. 28.5%; *p* < 0.001) and premature rupture of membranes (PROM) (37.6% vs. 19.1%; *p* < 0.001) than those in the non-BSI group. The median (interquartile range) duration of rupture membranes was 24.0 (10.0–48.0) h in the BSI group and 10.0 (3.0–27.0) h in the non-BSI group (*p* = 0.003). Furthermore, differences were found in the proportion of PROM >24 h before labor between the groups (*p* = 0.003). Patients in the BSI group were more likely to undergo vaginal examinations >5 times and cesarean section during labor (*p* < 0.05). The cervical ligation, placenta previa, retained products of conception, episiotomy or laceration, postpartum hemorrhage, epidural analgesia, or indwelling bladder catheterization did not significantly differ between the groups (*p* > 0.05).

**Table 1:** Demographic and clinical characteristics of the BSI and non-BSI groups.

**Characteristic**	**Total (*n =* 446)**	**BSI group (*n =* 85)**	**Non-BSI group (*n =* 361)**	***p* Value**
Age (years)	32.0 (29.0–34.0)	32.0 (29.0–35.0)	32.0 (29.0–34.0)	0.769
Pregestational BMI (Kg/m^2^)	22.73 (20.45–26.04)	23.44 (21.33–26.35)	22.55 (20.31–25.96)	0.070
Pregestational medical conditions ^a^ (diabetes and hypertension excluded)	218 (48.9)	41 (48.2)	177 (49.0)	0.895
Diabetes mellitus	128 (28.7)	22 (25.9)	106 (29.4)	0.523
Hypertension	75 (16.8)	12 (14.1)	63 (17.5)	0.460
Nulliparous	374 (83.9)	75 (88.2)	299 (82.8)	0.223
Multiple gestations	23 (5.2)	5 (5.9)	18 (5.0)	0.949
Gestation age at delivery (weeks)	39.14 (37.71–40.14)	39.14 (37.43–40.14)	39.14 (37.71–40.14)	0.864
Preterm birth	79 (17.7)	14 (16.5)	65 (18.0)	0.739
Mode of membranes rupture				<0.001
Spontaneous	143 (32.1)	40 (47.1)	103 (28.5)	
Artificial	303 (67.9)	45 (52.9)	258 (71.5)	
PROM	101 (22.6)	32 (37.6)	69 (19.1)	<0.001
Duration of ruptured membrane (hours)	13.5 (4.0–31.8)	24.0 (10.0–48.0)	10.0 (3.0–27.0)	0.003
PROM >24 h before labor	57 (12.8)	19 (22.4)	38 (10.5)	0.003
ART	51(11.4)	10 (11.8)	41 (11.4)	0.915
GBS positive ^b^	22 (17.1)	2 (6.9)	20 (20.0)	0.170
Cervical ligation	9 (2.0)	3 (3.5)	6 (1.7)	0.501
Placenta previa	18 (4.0)	4 (4.7)	14 (3.9)	0.966
Vaginal examinations >5 times	207 (46.4)	50 (58.8)	157 (43.5)	0.011
Mode of delivery				
Spontaneous vaginal	41 (9.2)	4 (4.7)	37 (10.2)	0.112
Assisted vaginal	7 (1.6)	1 (1.2)	6 (1.7)	>0.99
Cesarean during labor	132 (29.6)	33 (38.8)	99 (27.4)	0.038
Elective cesarean	266 (59.6)	47 (55.3)	219 (60.7)	0.364
Neonate birth weight (g)	3,260 (2,825–3,630)	3,310 (2,900–3,765)	3,255.0 (2,825.0–3,627.5)	0.351
Retained products of conception	19 (4.3)	4 (4.7)	15 (4.2)	>0.99
Episiotomy or laceration	48 (10.8)	5 (5.9)	43 (11.9)	0.107
Postpartum hemorrhage	15 (3.4)	3 (3.5)	12 (3.3)	>0.99
Epidural analgesia	137 (30.7)	32 (37.6)	105 (29.1)	0.124
Indwelling bladder catheterization	412 (92.4)	82 (96.5)	330 (91.4)	0.114

Data are presented as median (interquartile range) or *n* (%). ^a^Pregestational medical conditions: cardiac disease, liver disease, renal disease, endocrine disorders, mental health disorder/psychiatric disease, hematological conditions, autoimmune conditions, and cancer. ^b^317 patients were missing for GBS status. BSI, bloodstream infection; BMI, body mass index; ART, artificial reproductive technique; GBS, Group B *Streptococcus*.

### Infection features and laboratory test results

Table 2 presents a comparison of the temperature, timing of fever, duration of fever, and infection sources between the BSI and non-BSI groups. Peripartum women in the BSI group had higher body temperatures (median, 39.4 vs. 38.7°C; *p* < 0.001) and longer duration of fever (2.0 vs. 1.0 days; *p* < 0.001) than patients in the non-BSI group. The highest fever onset rate appeared postpartum, which accounted for up to 89% of the study population. Fever within 24 h postpartum was more common in the BSI group (42.3% vs. 26.3%; *p* = 0.006). Furthermore, the infection sources were recorded according to the initial related symptoms and laboratory test results. In addition, the most common cause in the BSI group was genital tract infections (71.8%; 61/85), followed by mixed infections (11.8%; 10/85), which were both significantly higher than those in the non-BSI group (71.8% vs. 26.3% and 11.8% vs. 4.7%; *p* < 0.05). Unknown infections accounted for the highest proportion in the non-BSI group (up to 37.1%).

**Table 2:** Infection features of BSI and non-BSI groups.

**Characteristic**	**Total (*n =* 446)**	**BSI group (*n =* 85)**	**Non-BSI group (*n =* 361)**	***p Value***
Temperature (°C)	38.8 (38.5–39.2)	39.4 (39.0–39.8)	38.7 (38.5–39.0)	<0.001
Duration of fever (days)	1.0 (1.0–2.0)	2.0 (1.0–3.0)	1.0 (1.0–2.0)	<0.001
Timing of fever				
Antepartum	16 (3.6)	2 (2.3)	14 (3.9)	0.722
Intrapartum	33 (7.4)	5 (5.9)	28 (7.8)	0.553
Postpartum	397 (89.0)	78 (91.8)	319 (88.3)	0.367
Time from delivery to postpartum fever ^a^				
within 24 h	117 (29.5)	33 (42.3)	84 (26.3)	0.006
24–48 h	132 (33.2)	26 (33.3)	106 (33.2)	0.986
48–72 h	96 (24.2)	14 (18.0)	82 (25.7)	0.152
after 72 h	52 (13.1)	5 (6.4)	47 (14.8)	0.051
COVID-19	3 (0.7)	0	3 (0.8)	>0.99
Infection types				
Genital tract	156 (35.0)	61 (71.8)	95 (26.3)	<0.001
Urinary tract or kidney	11 (2.4)	4 (4.7)	7 (2.0)	0.275
Respiratory tract	46 (10.3)	6 (7.0)	40 (11.1)	0.273
Gastrointestinal	4 (0.9)	0	4 (1.1)	>0.99
Breast	60 (13.4)	3 (3.5)	57 (15.8)	0.003
Skin, wound, and catheter infection	3 (0.8)	0	3 (0.8)	>0.99
Mixed infection	27 (6.0)	10 (11.8)	17 (4.7)	0.014
Other	4 (0.9)	0	4 (1.1)	>0.99
Unknown	135 (30.3)	1 (1.2)	134 (37.1)	<0.001
Preventive antibiotics	195 (43.7)	46 (54.1)	149 (41.3)	0.032
Duration of therapeutic antibiotic treatment (days) ^b^	5.0 (4.0–8.0)	9.0 (7.0–12.0)	5.0 (3.0–6.0)	<0.001

Data are presented as the median (interquartile range) or *n* (%). ^a^Time from delivery to postpartum fever in 78 postpartum BSI cases and 319 postpartum non-BSI cases. ^b^73 patients were missing because of the duration of therapeutic antibiotic treatment (days). COVID-19, Corona Virus Disease 2019.

**Figure 2:**
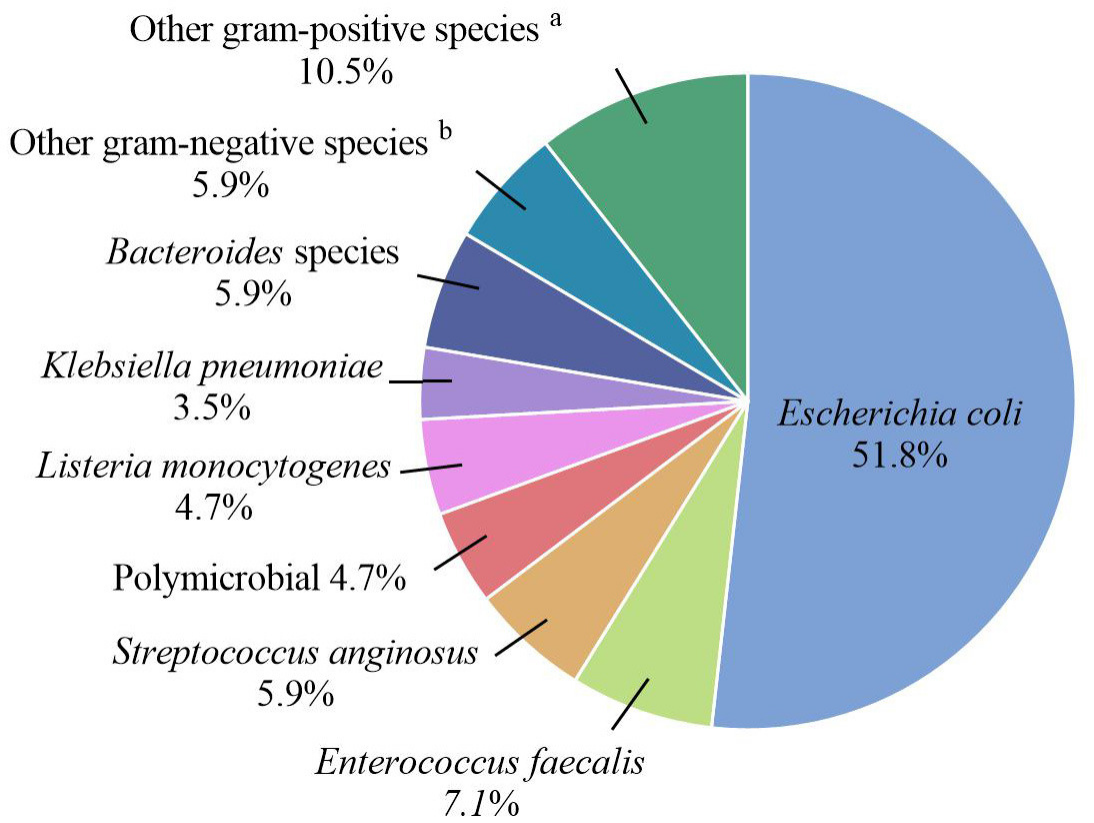
Pathogenic microorganisms of the BSI group. ^a^Other gram-positive species included *Gardnerella vaginalis, Group B streptococcus, Finegoldia magna, Clostridium perfringens*, and *Clostridium difficile*. ^b^Other gram-negative species included *Enterobacter aerogenes, Enterobacter cloacae, Morganella morganii*, and *Citrobacter koseri*.

*Escherichia coli* was the most frequently isolated pathogen among women with positive blood cultures (51.8%; 44/85), followed by *Enterococcus faecalis* (7.1%; 6/85) (Figure 2). Hence, we analyzed the antibiotic resistance patterns of these two most common pathogenic microorganisms isolated from blood cultures (Table 3). The results showed that the drug resistance rate of *E. coli* to levofloxacin was 31.2%, and the proportion of extended-spectrum β-lactamase (ESBL)-producing isolates was 38.6%. Of the six *E. faecalis* isolates, five (83.3%) were resistant to clindamycin, five (83.3%) to tetracycline, four (66.7%) to erythromycin, and two (33.3%) to levofloxacin. Preventive antibiotics were more likely to be used (54.1% vs. 41.3%; *p* = 0.032), and therapeutic antibiotics were used for a longer period (9.0 vs. 5.0 days; *p* < 0.001) in the BSI group than in the non-BSI group (Table 2). The therapeutic antibiotics used are shown in Supplementary Figure 1. The BSI group used penicillin and/or β-lactamase inhibitors most frequently, accounting for 67.1% (57/85) of the patients. Simultaneously, second-generation cephalosporins were most frequently used in the non-BSI group, accounting for 31.0% (112/361) (Supplementary Figure 1).

**Table 3:** Antibiotic resistance of the two most common pathogenic microorganisms of the BSI group.

**Organism**	**Penicillin resistance**	**Tetracycline resistance**	**Levofloxacin resistance**	**Erythromycin resistance**	**Clindamycin resistance**	**ESBL +**
*E. coli* (*n =* 44)	0 (0)	0 (0)	14 (31.2)	0 (0)	0 (0)	17 (38.6)
*Enterococcus faecalis* (*n =* 6)	0 (0)	5 (83.3)	2 (33.3)	4 (66.7)	5 (83.3)	0 (0)

ESBL, extended-spectrum β-lactamase. Data are presented by *n* (%).

### Maternal and neonatal outcomes

Table 4 presents the maternal and neonatal outcomes in the study population. The proportions of peripartum women admitted to the ICU or the length of stay in the ICU did not differ significantly between the two groups (*p* > 0.05). Eleven cases (12.9%) in the BSI group progressed to sepsis; however, no patients in the non-BSI group did. We analyzed the outcomes of neonates, including 43 born to mothers with BSI before delivery or within the first 24 h postpartum and 134 born to mothers with non-BSI with initial fever in the same period. Neonates in the BSI group had a lower gestational age at delivery compared to those in the non-BSI group (*p* = 0.045). However, no differences in the percentage of preterm births were observed between the two groups (*p* = 0.194). The incidence of an Apgar score of ≤ 7 at 1 min was higher in the BSI group than in the non-BSI group (14.0% vs. 3.7%, *p* = 0.040). The proportion of neonates who developed bacteremia or sepsis was 27.9% in the BSI group and 29.1% in the non-BSI group, with no significant difference (*p* = 0.880). Of the 18 neonates with sepsis, eight had positive blood cultures. Results from blood cultures in neonates and mothers are presented in Supplementary Table 1. The microbiological profiles of 50% of neonates matched those of their mothers. Univariate analysis revealed significant differences in premature birth, multiple gestation, and preterm premature rupture of membranes with regard to neonatal bacteremia/sepsis. Multivariate analysis identified premature birth as a risk factor, increasing the incidence of neonatal bacteremia/sepsis by seven times (adjusted odds ratio [aOR]: 7.042, 95% confidence interval [CI]: 2.611–18.991; *p* < 0.001).

**Table 4:** Maternal and neonatal outcomes in BSI group and non-BSI group.

**Outcomes**	**Total (*n =* 446)**	**BSI group (*n =* 85)**	**Non-BSI group (*n =* 361)**	***p* Value**
Maternal outcomes				
ICU admission	16 (3.6)	6 (7.1)	10 (2.8)	0.112
ICU length of stay (days)	3.5 (3.0–4.8)	3.5 (3.0–4.8)	3.5 (2.0–5.0)	0.738
Sepsis	11 (2.5)	11 (12.9)	0	<0.001
Neonatal outcomes ^a^				
Gestation age at delivery (weeks)	39.3 (37.2–40.1)	38.7 (36.0–40.0)	39.4 (37.4–40.3)	0.045
Preterm birth	37 (20.9)	12 (27.9)	25 (18.7)	0.194
Apgar score of ≤ 7 at 1 min	11 (6.2)	6 (14.0)	5 (3.7)	0.040
Apgar score of ≤ 7 at 5 min	3 (1.7)	2 (4.7)	1 (0.7)	0.147
Umbilical cord pH <7.0	2 (2.5)	1 (4.8)	1 (1.7)	0.459
NICU admission	80 (45.2)	21 (48.8)	59 (44.0)	0.582
NICU length of stay (days)	10.0 (6.0–14.0)	11.0 (7.0–22.5)	8.0 (6.0–13.0)	0.121
Neonatal bacteremia/sepsis	51 (28.8)	12 (27.9)	39(29.1)	0.880
Blood culture samples obtained at less than 7 days of age ^b^	48 (94.1)	12 (100)	36 (92.3)	>0.99

^a^ Neonatal outcomes were presented for the 43 neonates born to mothers with BSI before delivery or within the first 24 h postpartum and 134 born to mothers with negative blood cultures with initial fever in the same period. ^b^ Fifty one neonates received blood cultures, of which 12 from maternal BSI group and 39 from maternal non-BSI group. ICU, intensive care unit; NICU, neonatal intensive care unit.

### Risk factors of postpartum bloodstream infections

In total, 397 (89.0%) patients with postpartum fever who underwent blood culture tests were reported, including 78 and 319 postpartum patients in the BSI and non-BSI groups, respectively. Most cases of fever were postpartum fever cases. Different risk factors may be associated with antepartum, intrapartum, and postpartum fever (Ngonzi et al., 2018; Megli and Coyne, 2022). Risk factors for antepartum or intrapartum BSI were not analyzed because of the limited number of cases of antepartum and intrapartum fever. The univariate analysis results are presented in Supplementary Table 2. In the multivariable logistic regression analysis (Table 5), PROM (aOR: 2.234; 95% CI: 1.214–4.110; *p* = 0.010), fever ≥ 38.9 °C (102°F) (aOR: 9.452; 95% CI: 4.702–18.999; *p* < 0.001), and fever within 24 h after delivery (aOR: 2.347; 95% CI: 1.302–4.232; *p* = 0.005) were independent risk factors for developing postpartum BSI after adjustment for parity, pre-gestational BMI, pre-gestational medical conditions (diabetes and hypertension excluded), gestation age at delivery, and use of preventive antibiotics.

**Table 5:** Unadjusted and adjusted odds ratios of women with postpartum BSI, OR, odds ratio.

**Characteristic**	**Unadjusted OR (95% CI)**	Adjusted OR^*^(95% CI)
PROM	2.540 (1.490–4.328)	2.234 (1.214–4.110)
fever of ≥38.9°C (102°F)	9.089 (4.624–17.865)	9.452 (4.702–18.999)
Fever within 24 h after delivery	2.052 (1.227–3.429)	2.347 (1.302–4.232)

^*^Adjusted for parity, pre-gestational BMI, pre-gestational medical conditions (diabetes mellitus and hypertension excluded), gestational age at delivery, and antibiotic prophylaxis.

## Discussion

### Principal findings

This retrospective cohort study found differences between the BSI group and non-BSI in modes of membrane rupture, duration of membrane rupture, delivery methods, and whether the vaginal examination was performed more than five times. Patients in BSI experienced more severe fever, typically within the first 24 h postpartum, and had longer-lasting fever compared to those in the non-BSI group. Genital tract infections were more common in patients with BSI, followed by mixed, respiratory, and urinary tract infections, whereas 37.1% of infections in non-BSI patients were of unknown origin. Patients with BSI were often treated with penicillin and/or β-lactamase inhibitors (67.1%), while non-BSI patients were predominantly treated with second-generation cephalosporins (31.0%). The BSI group had higher rates of maternal sepsis and neonates with Apgar scores ≤ 7 at 1 min compared to the non-BSI group. Patients with BSI received prophylactic antibiotics more frequently and for a longer duration than patients without BSI. Furthermore, PROM, fever ≥38.9 °C (102°F), and fever within 24 h after delivery were independent risk factors for postpartum BSI.

### Morbidity

Previous studies of maternal BSI have reported an incidence rate over the decade ranging from 21/10,000 in the United States (2009–2016) (Wilkie et al., 2019), 27/10,000 in Canada (2010–2018) (Mohn et al., 2024), 10/10,000 in China (2013–2022) (Guo et al., 2023), and 37/10,000 in the United States (2014–2018) (Mohn et al., 2024). The wide range of incidences may be related to differences in the study design, survey period, and background population characteristics. However, the incidence rate identified in our study (44/10,000) was slightly higher than that reported in most of the aforementioned studies. In addition, the higher morbidity observed in our study may be because our study was conducted at a single center, and the study institution was a critical maternity care center. Currently, there is no uniform standard for indicating blood culture in obstetric BSI (Evans et al., 2021; Shields et al., 2023). This lack of standardization leads to variations in collection methods and timing across regions, potentially contributing to significant differences in peripartum BSI morbidity. In addition, comorbid medical conditions such as gestational diabetes mellitus and gestational autoimmune disorders were highly prevalent in this setting, which may increase the susceptibility to infections. Furthermore, our study had different inclusion and exclusion criteria compared to similar research; we excluded cases of fetal anomalies but did not exclude patients who used antibiotics or those who were preterm, aiming to accurately represent the clinical characteristics of febrile peripartum women. We excluded fetal anomalies because maternal infections such as TORCH (*Toxoplasma gondii*, other, rubella virus, cytomegalovirus, and herpes simplex virus) can have teratogenic effects leading to congenital anomalies, influencing microbial characteristics and neonatal outcomes (Megli and Coyne, 2022). Furthermore, the investigation period was relatively brief (2018–2023), and the coronavirus disease 2019 pandemic increased the risk of infection. In addition, the higher morbidity rate in our study may have resulted from various factors, including study design and methodology, demographic factors, and survey period, limiting the generalizability of the results. Future research should address these factors more comprehensively and in diverse populations.

### Risk factors

Previous studies have noted that 47.4% of patients with postpartum BSI have PROM, which is considered a risk factor for postpartum BSI (Zou et al., 2021). Several studies have demonstrated that obesity, gestational diabetes, multiparity, fever during labor, and fever ≥38.9°C (102°F) increased the risk of maternal BSI during the peripartum period (Surgers et al., 2013; Easter et al., 2017). In addition, PROM and preterm birth increase the risk of neonatal infection (Guo et al., 2023). Notably, studies on the risk factors of peripartum BSI are scarce and show significant heterogeneity, possibly because of differences in BSI definition, study populations, sample sizes, and research designs. In this study, we identified PROM, fever ≥38.9°C (102°F), and fever within 24 h postpartum as independent risk factors for postpartum BSI. PROM may lead to chorioamnionitis, with the risk increasing with prolonged membrane rupture. The incidence of PROM and PROM >24 h before labor was significantly higher in the BSI group than in the non-BSI group. In addition, the incidence of BSI increased with prolonged PROM. Notably, spontaneous membrane rupture was more common in the BSI group than in the non-BSI group. Furthermore, careful monitoring of labor and vital signs and reducing the frequency of vaginal examinations can help prevent maternal BSI for patients with PROM.

There is an association between fever severity and maternal BSI, with significant maternal fever defined as 39.0°C (102.2°F) (Higgins et al., 2016; Molina et al., 2015). Molina et al. (2015) found that a maximum fever exceeding 38.9°C (102 °F) was closely related to the odds of peripartum BSI (aOR: 3.37; 95% CI: 1.61–7.06; *p* = 0.001). We observed that in the population of postpartum febrile women, 85.9% (67/78) of women in the BSI group had a fever of >38.9°C (102°F); however, the proportion in the non-BSI group was only 40.1% (128/319). This was consistent with the findings from Molina's study (Molina et al., 2015). We further reported that maternal temperatures >38.9°C (102°F) were significantly associated with postpartum BSI. These findings suggest that patients with risk factors should be closely monitored after delivery, and BSI should be highly suspected when maternal body temperatures exceed 38.9°C (102°F). Furthermore, fever within 24 h of delivery was identified as an independent risk factor for BSI. A maternal temperature >38.9°C (102°F) within 24 h of delivery this timeframe can serve as an early warning for BSI, warranting timely initiation or escalation of antibiotics, even in the absence of other risk factors.

### Infection sources

Several studies have shown that most cases of antepartum sepsis are caused by genitourinary infections and that pyelonephritis is the most common cause of infection in antepartum non-obstetric hospitalizations (Hensley et al., 2019; Knowles et al., 2015; Bauer et al., 2013). The majority of intrapartum and postpartum sepsis occur secondary to genitourinary or respiratory infections (Hensley et al., 2019; Knowles et al., 2015). However, a retrospective case-control study in Italy found that respiratory and urinary tract infections were the most common sources of infection in antepartum sepsis, and genital tract infections were the most common sources of infection in postpartum sepsis (Ornaghi et al., 2022). Furthermore, Guo et al. (2023) found that the urinary and reproductive tracts were the predominant sources of postpartum BSI, accounting for 13% and 28%, respectively, consistent with previous studies. The highest onset rate was in the postpartum period (89.0%; 397/446), and the main source of infection was genital tract infections, followed by mixed, respiratory, and urinary tract infections for the BSI cases in our study.

Notably, non-infectious peripartum fever is common, particularly during labor, leading to either overtreatment with antibiotics or undertreatment of patients with suspected non-infectious fever (Riley et al., 2011; Zhao et al., 2022). Approximately a third of the population in the non-BSI group had no specific symptoms and lacked identifiable sources of infection. However, not all causes of fever during labor are associated with bacterial infections, according to some studies. For example, epidural anesthesia has been associated with intrapartum hypothermia, thought to be caused by non-infectious inflammation (Higgins et al., 2016; Sharma et al., 2014). Additionally, exposure to prostaglandins, hyperthyroidism, dehydration, and excess ambient heat are other causes of non-bacterial maternal fever (Higgins et al., 2016). In this retrospective study, 37.1% of the non-BSI population had an unexplained fever, which may be because of a recording bias or this portion of the population was indeed non-infectious. These findings may assist clinicians in achieving an optimal balance between early diagnosis and treatment of febrile peripartum women while reducing the non-essential use of antibiotics.

Must there be BSI in a patient with sepsis? The answer is no. Patients with sepsis may lack a source of infection or positive blood cultures (Kinasewitz et al., 2004; Plante et al., 2019). Ornaghi et al. (2022) reported that the source of infection could not be identified in 18.3% of patients, and 32.4% had negative blood cultures. In addition, neither the source of infection nor the causative organism was identified in 10% of cases. Furthermore, Acosta et al. reported similar findings, in which the source of infection could not be identified in 26% of cases, blood cultures were negative in 36.2%, and 16.4% had neither a source of infection nor an identified causative microorganism (Acosta et al., 2014). Notably, BSI causes sepsis in only 25–30% of cases, with no definitive test to distinguish sepsis from BSI; therefore, a comprehensive clinical assessment is required to differentiate between the two conditions (Huerta and Rice, 2019). We found that the proportion of patients with sepsis in the BSI group was 12.9%; however, there were none in the non-BSI population. There may still be cases of BSI and sepsis that were not recorded because of normal temperatures or neglected symptoms. Another possible explanation is that our study population was limited to patients hospitalized for delivery, and no follow-up was performed.

### Pathogenic microorganisms and antibiotics

Under certain conditions, the normal flora of the genital tract may spread upward. Common pathogenic bacteria of genital tract infections include *E. coli*, Group B *Streptococcus* (GBS), *Klebsiella, Enterococcus*, and anaerobes (Knowles et al., 2015; Acosta et al., 2014). *E. coli* was the most common bacterium causing BSI, which is consistent with the results of other studies (Knowles et al., 2015; Ornaghi et al., 2022; Abir et al., 2017; Vasco et al., 2019). Additionally, Knowles et al. reported that the second most common pathogen was GBS (Knowles et al., 2015). In contrast, of the 40 cultured microorganisms from the placental swabs in our study, five were GBS, and none of them spread upwards and developed into BSI. In 12 BSI cases with placental swab cultures, six BSI cases were positive for *E. coli*, of which five were positive for *E. coli* in blood culture, and one had puerperal sepsis. Furthermore, two cases were positive for *Listeria monocytogenes* after placental swab cultures, both of which were complicated by BSI and sepsis caused by *L. monocytogenes*. Three women in our cohort had positive blood cultures for *L. monocytogenes*, with an incidence of 15.9 per 100,000, consistent with other studies (Khsim et al., 2022; Zhang et al., 2023; Ke et al., 2022). Due to the limited sample size, we did not analyze the correlation and causality of the co-culture of placental swabs and blood. However, microbial culture from placental swabs cannot be interpreted as indicative of genital tract infection; the gold standard for determining maternal-fetal interface infections is generally considered to be placental histopathology (Goldstein et al., 2020). Because only 27 placentas underwent pathological examination, identifying two cases of chorioamnionitis, both in the BSI group, whether uterine microorganisms can cross the maternal-fetal interface to cause maternal-fetal infections needs to be validated in a larger sample population.

A comprehensive investigation of perinatal infection with *L. monocytogenes* would be appropriate for future studies. “*Chinese Experts Consensus on Prevention of Perinatal Group B Streptococcal Disease,”* published in 2021, proposed that all pregnant women in China should undergo antepartum screening for GBS at 35–37 weeks of gestation for the first time. The 317 pregnant women enrolled in our study between 2018 and 2023 did not undergo the GBS screening; however, 129 pregnant women did, with 22 (17.1%) testing positive for GBS colonization, thus preventing further analysis of the effects of GBS status on maternal BSI. Overall, the GBS colonization rate was 17.9–18.0% (Kwatra et al., 2016; Russell et al., 2017). Notably, a meta-analysis of 30 Chinese studies conducted between 2000 and 2018, including 44,716 pregnant women, revealed a GBS colonization rate of 11.3% among Chinese women (Ding et al., 2020). The variation in colonization rates, both locally and internationally, may be attributed to differences in sampling sites, methods, gestational age of testing, and testing techniques. However, our population's GBS colonization rate is closely aligned with the global average but higher than the local rate, likely because of the broader implementation of the GBS screening.

Previous studies have demonstrated that severe infections during pregnancy and labor are the main risk factors for adverse perinatal outcomes. Blauvelt et al. (2021) found that sepsis during pregnancy is associated with placental dysfunction. A review of previous literature on peripartum women with suspected or confirmed infections revealed that a third of newborns had adverse outcomes (Baguiya et al., 2021). Another study conducted in the United Kingdom also reported similar results: the rates of maternal ICU and neonatal ICU (NICU) admissions were 31.2% and 42.3%, respectively (Acosta et al., 2014). We did not observe any maternal and neonatal deaths in the population; however, in the BSI group, 20% of the women had severe adverse outcomes, 12.9% developed sepsis, and 7.1% were admitted to the ICU. Of these, 66.7% were admitted because of sepsis or BSI. Moreover, no patients with sepsis were recorded in the non-BSI group, while 10 patients were admitted to the ICU for medical or surgical comorbidities. Additionally, 48.8% (21/43) of neonates in the BSI group were admitted to the NICU, and 14.0% (6/43) had an Apgar score of ≤ 7 at 1 min. Furthermore, the rates of neonatal sepsis did not differ between the groups, with no significant difference in the incidence of premature infants and the time of retention of blood culture. Similarly, 94.1% of the blood cultures were taken within 7 days of birth, indicating that most of the neonatal sepsis was of early onset. Although maternal BSI is associated with neonatal infection, and intra-amniotic infection and chorioamnionitis have been confirmed to be associated with early-onset sepsis (EOS) (Shane et al., 2017), there is no difference in the incidence of early-onset neonatal sepsis between the BSI and the non-BSI groups. This may relate to the high rates of postpartum infection after the neonate is delivered; at our institution neonatal blood cultures are not collected in the setting of maternal infection; the placental defenses against infection remain intact in the setting of intrapartum bacteremia. Eight neonates had positive blood cultures among the 18 with sepsis. The microbiological profiles of 50% of neonates matched those of their mothers, suggesting that maternal bacteremia may have crossed the maternal-fetal interface, resulting in neonatal infection. However, further data are needed to establish this connection. The limited number of episodes and observed variables, in addition to unrecorded factors, such as maternal comorbidities and gestational age, may have affected the outcomes. Nevertheless, the timely prevention, early diagnosis, and treatment of infections during pregnancy can improve maternal and neonatal outcomes. In 2023, the Society for Maternal-Fetal Medicine recommended empiric broad-spectrum antibiotic therapy within 1 h of identifying infection in pregnant women or postpartum patients with septic shock or suspected sepsis (Grade 1C) (Shields et al., 2023). Hence, patients with isolated fever would benefit from antibiotic treatment during labor despite potential non-bacterial sources of infection (Bank et al., 2022). The population with BSI in this study used antibiotics prophylactically and for longer durations. Based on our findings, uncovering the outcomes of the population receiving antibiotic prophylaxis remains a challenge; therefore, further research should assess the efficacy of prophylactic antibiotics.

Our results showed that 31.2% of *E. coli* strains were resistant to levofloxacin, while 38.6% were identified as ESBL-producing bacteria. Similar studies on maternal BSI caused by *E. coli* reported that the drug resistance rate of *E. coli* against levofloxacin ranges from 16%−21% (Guo et al., 2023; Wen et al., 2021), and the proportion of ESBL-producing isolates ranges from 47.6%−50% (Guo et al., 2023; Wilkie et al., 2019). Differences in *E. coli* resistance may be related to contingency, epidemiological, and geographical characteristics or increased antibiotic exposure during pregnancy.

We did not identify penicillin- or vancomycin-resistant *E. faecalis* isolates from blood cultures. In addition, ampicillin or vancomycin monotherapy remains effective for BSI caused by *Enterococci* (Rosselli Del Turco et al., 2021). Hence, Obstetricians must strictly control the indications for vancomycin and rationally select antibiotics. Furthermore, penicillin and/or β-lactamase inhibitors were the most commonly used antibiotics in the BSI group, which was consistent with the antibiotic resistance patterns. In contrast, penicillin and second-generation cephalosporins were most commonly used in the non-BSI group, indicating a preventive effect of penicillin and second-generation cephalosporins against BSI; however, this effect may be influenced by local antibiotic usage patterns. Based on the results of microbial antibiotic resistance from this study, previous empirical antibiotic protocols should be re-evaluated. Penicillins or second- and third-generation cephalosporins may be considered as the first-line empirical antibiotic choice for pregnant women with suspected urinary and genital tract infections or suspected BSI. Presumed sources of infection, microbial species, and local antibiotic resistance patterns affect the choice of empirical antibiotics. Hence, the evolution of antibiotic resistance patterns may be clinically significant in determining antibiotic selection in febrile women during the peripartum period.

### Strengths and limitations

This study is one of the few studies on maternal BSI during delivery hospitalization from a microbiological perspective. We analyzed the demographic and clinical characteristics of 446 peripartum febrile women, with a sample size significantly larger than that of previous studies (Easter et al., 2017; Molina et al., 2015). In addition, we performed a detailed chart review covering all medical records in our institution over 5 years, providing relatively complete and accurate data on patients admitted for delivery and a comprehensive description of the clinical characteristics, microbiological features, antibiotic resistance, use of antibiotics, and risk factors for maternal peripartum BSI. Notably, we identified PROM, fever ≥38.9°C (102°F), and fever within 24 h after delivery as independent risk factors for postpartum BSI, which has received little attention.

However, the study had some limitations. This study was conducted at a single center, which limits the generalizability of the results at the regional or national level. The 95% CIs of some aORs were too wide, leading to a low certainty of the results. Moreover, our research may not have included all eligible febrile women because of missing data. It is also possible that false-negative culture results were obtained in the non-BSI group. Furthermore, the outcomes and clinical significance of over-testing and overtreatment in the non-BSI population were not assessed. Therefore, a multicenter, large-scale study would be appropriate to fully understand the underlying intricacies of the epidemiological, clinical, microbiological, and antibiotic molecular resistance mechanisms. These results provide a valuable framework for the development of effective infection control strategies.

## Data Availability

The original contributions presented in the study are included in the article/Supplementary material, further inquiries can be directed to the corresponding author.
