# Supplementary material for: Clinical characteristics associated with peripartum maternal bloodstream infection
Source: Front Microbiol. 2024 Nov 13;15:1454907. doi: 10.3389/fmicb.2024.1454907 (PMC11599977; doi:10.3389/fmicb.2024.1454907)
Supplement: Supplementary file 1 [file Data_Sheet_1.pdf]

Supplementary Material

Supplementary Figures and Tables

Supplementary Figure 1 Type of therapeutic antibiotics administered among BSI group (**A**) and non-BSI group (**B**).

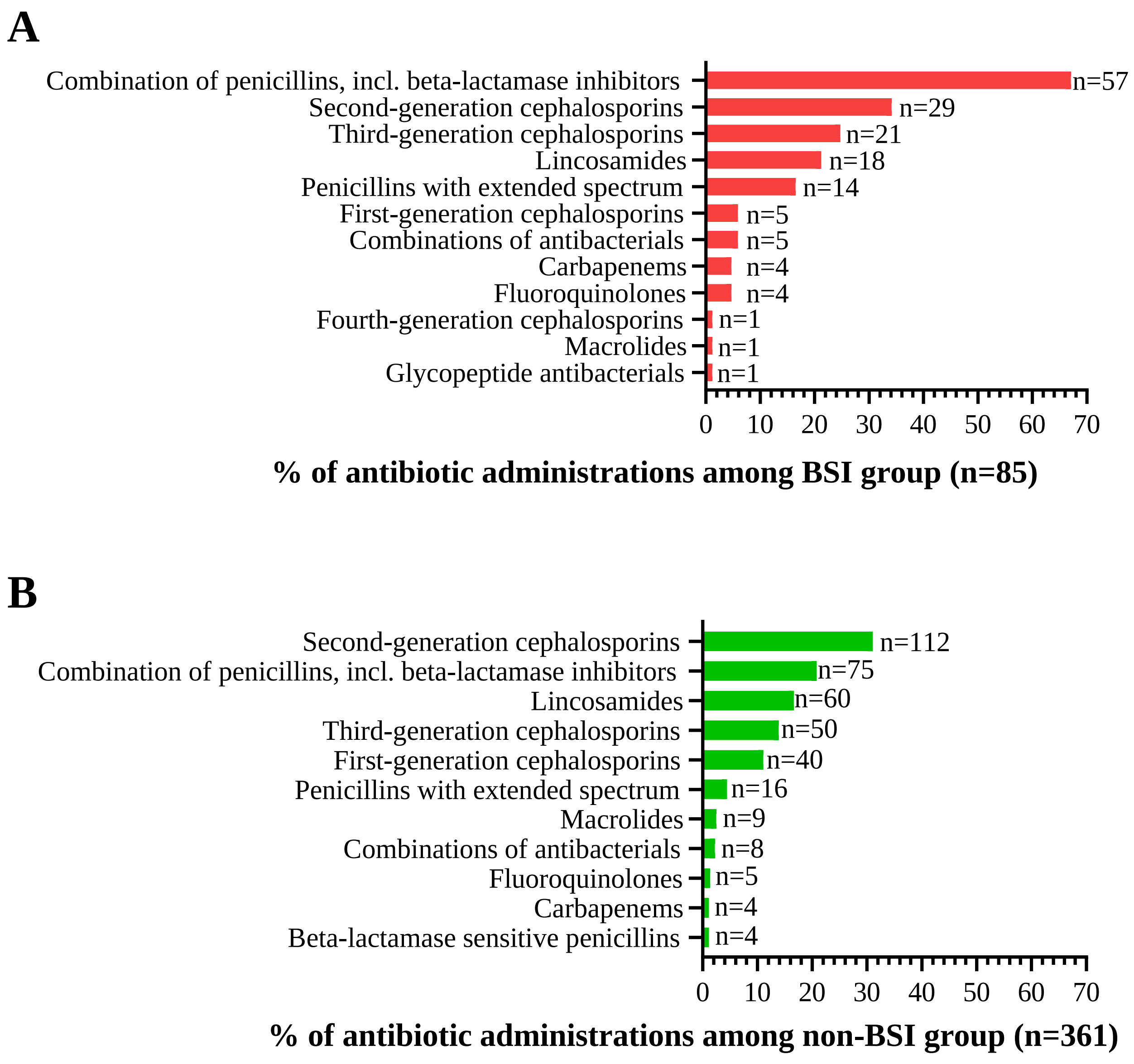

Supplementary Table 1 Blood cultures in neonates with sepsis and the mothers.

Neonates No.	Isolated Bacteria from Neonates	Isolated Bacteria from Mothers
1	Group A *Streptococcus*	Group A *Streptococcus*
2	Group A *Streptococcus*	Group A *Streptococcus*
3	*Listeria monocytogenes*	*Listeria monocytogenes*
4	*Listeria monocytogenes*	*Listeria monocytogenes*
5	*Escherichia coli*	*Lactobacillus salivarius*
6	*Listeria monocytogenes*	-
7	*Listeria monocytogenes*	-
8	*Klebsiella pneumoniae*	-

Supplementary Table 2 Univariate analysis of women with postpartum BSI.

Characteristic	Total (n=397)	BSI Group (n=78)	Non-BSI Group (n=319)	*p* Value
Age (years)	32.0 (29.0-35.0)	31.5 (29.0-35.0)	32.0 (29.0-34.0)	0.944
Pregestational BMI (Kg/m2)	22.9 (20.6-26.1)	23.4(21.3-26.4)	22.8 (20.5-26.0)	0.222
Pregestational medical conditions (diabetes and hypertension excluded)	195 (49.1)	38 (48.7)	157 (49.2)	0.937
Diabetes mellitus	110 (27.7)	21 (26.9)	89 (27.9)	0.863
Hypertension	71 (17.9)	12 (15.4)	59 (18.5)	0.520
Nulliparous	333 (82.4)	70 (89.7)	263 (82.4)	0.116
Multiple gestations	19 (4.8)	3 (3.8)	16 (5.0)	0.890
Gestation age at delivery (weeks)	39.1 (37.7-40.1)	39.4 (37.7-40.2)	39.0 (37.7-40.9)	0.509
Mode of membranes rupture				<0.001
Spontaneous	125 (31.5)	37 (47.4)	88 (27.6)	
Artificial	273 (68.5)	41 (52.6)	231 (72.4)	
PROM	93 (23.4)	30 (38.0)	63 (19.7)	<0.001
PROM >24 h before labor	54 (13.6)	19 (24.4)	35 (11.0)	0.002
ART	46 (11.6)	8 (10.3)	38 (11.9)	0.682
GBS positive [a]	17 (16.3)	2 (7.7)	15 (19.2)	0.284
Cervical ligation	8 (2.0)	2 (2.6)	6 (1.9)	>0.99
Placenta previa	18 (4.5)	4 (5.1)	14 (4.4)	>0.99
vaginal examinations >5 times	174 (43.8)	47 (60.3)	127 (39.8)	0.001
Mode of delivery				
Spontaneous vaginal	27 (6.8)	3 (3.9)	24 (7.5)	0.248
Assisted vaginal	5 (1.3)	0	5 (1.6)	0.588
Cesarean during labor	112 (28.2)	31 (39.7)	81 (25.4)	0.012
Elective cesarean	253 (63.7)	44 (56.4)	209 (65.5)	0.134
Neonate birth weight (g)	3260 (2840-3630)	3310 (2985-3765)	3252 (2814-3628)	0.168

Retained products of conception	13 (3.3)	3 (3.8)	10 (3.1)	>0.99
Episiotomy or laceration	32 (8.1)	3 (3.8)	29 (9.1)	0.127
Postpartum hemorrhage	10 (2.5)	1 (1.3)	9 (2.8)	0.708
Epidural analgesia	116 (29.2)	30 (38.5)	86 (27.0)	0.045
Indwelling bladder catheterization	376 (94.7)	76 (97.4)	300 (94.0)	0.359
Preventive antibiotics [a]	188 (47.4)	44 (56.4)	144 (45.1)	0.074
Duration of therapeutic antibiotic treatment (days) [b]	5.0 (4.0-8.0)	9.0 (6.5-11.5)	4.0 (3.0-6.0)	<0.001
Temperature higher than 38.9℃	195 (49.1)	67 (85.9)	128 (40.1)	<0.001

[a] The indications for preventive antibiotic use in our institution were as follows: a. Elective cesarean section or emergency cesarean section; b. Patients with preterm premature rupture of membranes (gestation <34 weeks) should receive intravenous antibiotics to prevent infection, and those with term premature rupture of membranes for more than 12 hours should be given oral antibiotics to prevent infection; c. Antibiotics should be used prophylactically for GBS under any of the following conditions: (1) A history of neonatal GBS disease; (2) A positive GBS screen in the current pregnancy; (3) GBS bacteriuria during the current pregnancy. If the GBS screening results for the current pregnancy are not yet available but there is at least one high-risk factor present: inevitable preterm birth, preterm premature rupture of membranes, membrane rupture ≥18 hours, or intrapartum fever ≥38 ℃, a broad-spectrum antibiotic that covers GBS should be used; d. Prevention of bacterial endocarditis infection during childbirth: Consider prophylactic antibiotic use for high-risk patients (including those with cyanotic heart disease or those with artificial heart valves, or both) during vaginal delivery; e. A single dose of antibiotic at the time of repair is used in the setting of obstetric anal sphincter injuries (OASIS); f. Patients with lengthy surgical procedures or those who experience excessive blood loss should receive an additional intraoperative dose of the same antibiotic given (Coleman et al., 2018, van Schalkwyk and Van Eyk, 2017, Society of Perinatal Medicine, Chinese Medical Association, 2021).

[b] Missing for each variable was the following: GBS=293, Duration of therapeutic antibiotic treatment (days)=64.

References

Coleman, J., Murtha, A., Silverman, N. S. & Bulletin, A. C. P. (2018). Use of Prophylactic Antibiotics in Labor and Delivery. *Obstetrics and Gynecology,* 132**,** E103-E119. doi:10.1097/aog.0000000000002833.

Society of Perinatal Medicine, Chinese Medical Association. (2021). Chinese experts consensus on prevention of perinatal Group B Streptococcal disease. *Chinese Journal of Perinatal Medicine,* 24**,** 561-6

Van Schalkwyk, J. & Van Eyk, N. (2017). No. 247-Antibiotic Prophylaxis in Obstetric Procedures. *Journal of Obstetrics and Gynaecology Canada*, 39**,** e293-e299. doi:https://doi.org/10.1016/j.jogc.2017.06.007.

.
